# Cloning, Characterization and Expression Pattern Analysis of a Cytosolic Copper/Zinc Superoxide Dismutase (*SaCSD1*) in a Highly Salt Tolerant Mangrove (*Sonneratia alba*)

**DOI:** 10.3390/ijms17010004

**Published:** 2015-12-22

**Authors:** Enze Yang, Shanze Yi, Fang Bai, Dewei Niu, Junjie Zhong, Qiuhong Wu, Shufang Chen, Renchao Zhou, Feng Wang

**Affiliations:** 1 College of Pharmacy, Jinan University, Guangzhou 510632, China; yangenze@foxmail.com (E.Y.); yishanze317@163.com (S.Y.); 18739977531@163.com (D.N); 18825084106@163.com (J.Z.); qiuhong6506@126.com (Q.W.); 2 Guangdong Provincial Key Laboratory of Pharmacodynamic Constituents of TCM and New Drugs Research, Jinan University, Guangzhou 510632, China; 3 School of Life Sciences, Shenzhen University, Shenzhen 518060, China; 15016733519@163.com; 4 State Key Laboratory of Biocontrol and Guangdong Provincial Key Laboratory of Plant Resources, School of Life Sciences, Sun Yat-Sen University, Guangzhou 510275, China; chsuf@mail.sysu.edu.cn

**Keywords:** *Sonneratiaalba*, copper/zinc superoxide dismutase, protein expression, activity and stability, salt stress

## Abstract

Mangroves are critical marine resources for their remarkable ability to tolerate seawater. Antioxidant enzymes play an especially significant role in eliminating reactive oxygen species and conferring abiotic stress tolerance. In this study, a cytosolic copper/zinc superoxide dismutase (*SaCSD1*) cDNA of *Sonneratia alba*, a mangrove species with high salt tolerance, was successfully cloned and then expressed in *Escherichia coli* Rosetta-gami (designated as SaCSD1). *SaCSD1* comprised a complete open reading frame (ORF) of 459 bp which encoded a protein of 152 amino acids. Its mature protein is predicted to be 15.32 kDa and the deduced isoelectric point is 5.78. *SaCSD1* has high sequence similarity (85%–90%) with the superoxide dismutase (*CSD*) of some other plant species. *SaCSD1* was expressed with 30.6% yield regarding total protein content after being introduced into the pET-15b (*Sma* I) vector for expression in Rosetta-gami and being induced with IPTG. After affinity chromatography on Ni-NTA, recombinant *SaCSD1* was obtained with 3.2-fold purification and a specific activity of 2200 U/mg. *SaCSD1* showed good activity as well as stability in the ranges of pH between 3 and 7 and temperature between 25 and 55 °C. The activity of recombinant *SaCSD1* was stable in 0.25 M NaCl, Dimethyl Sulphoxide (DMSO), glycerol, and chloroform, and was reduced to a great extent in β-mercaptoethanol, sodium dodecyl sulfate (SDS), H_2_O_2,_ and phenol. Moreover, the *SaCSD1* protein was very susceptive to pepsin digestion. Real-time Quantitative Polymerase Chain Reaction (PCR) assay demonstrated that *SaCSD1* was expressed in leaf, stem, flower, and fruit organs, with the highest expression in fruits. Under 0.25 M and 0.5 M salt stress, the expression of *SaCSD1* was down-regulated in roots, but up-regulated in leaves.

## 1. Introduction

Mangroves play an important role in protecting coastal areas by buffering erosion from waves and thus reducing damage from typhoons and sequestering carbon [1,2]. *Sonneratia alba*, one of the most widespread and salt-resistant mangrove trees, grows in the swampy salt water zones of tropical and subtropical coasts [3]. It can adapt to salinities varying from fresh water to sea water [4,5]. Although the increasing ecological importance of mangroves is known, the molecular mechanisms elucidating their adaptation to extremely high saline intertidal habitats remain elusive. Abiotic external stress factors, such as drought, salinity, strong light intensity, extreme temperature, and heavy metals, may result in oxidation stress with the accumulation of Reactive oxygen species (ROS), causing huge inhibition in photosynthesis and great cellular damage [6]. The resulting excessive ROS from the extreme salinity of the intertidal environments must be removed efficiently [6,7,8]. superoxide dismutases (SODs) function as the first line of defense in the enzymatic pathway of defense against free oxygen radicals [9]. In plants, SODs can be divided into three distinct groups based on different metal ions: copper/zinc SOD (Cu/Zn-SOD), manganese SOD (Mn-SOD), and iron SOD (Fe-SOD) [10]. Mn-SOD only exists in mitochondria and Fe-SOD only exists in chloroplasta, while CSD is the most abundant SOD in plants, and has been mainly located in the cytosol. In general, the activity of antioxidant enzymes and plant resistance strength has a positive correlation[11]. It has been reported that the SOD activities of field-grown mangroves are nearly 40 times higher than those in some common crop plants such as the pea [12]. Moreover, certain antioxidative enzymes against activated oxygen under salt stress, such as ascorbic peroxidase (APX), guaiacol peroxidase (GPX), glutathione reductase (GR), and superoxide dismutase (SOD), were found to be elevated in one mangrove species, *Bruguiera parviflora* [13].

Although CSDs have been cloned from a number of plant species, such as sweet potato [14], pea [15], and rice [16], there have been limited reports in two mangrove species, *Avicennia marina* and *Bruguiera gymnorhiza* [6], due to insufficient transcriptomic and genomic data in the public databases. Moreover, characterization of CSDs has not been studied in highly salt-tolerant mangrove plants.

SOD is widely used in clinical therapy, medicines, food, and cosmetics as an efficacy factor or as an additive [17,18,19,20,21], such as in some beer and revitalizers. For instance, exogenous SOD added in a yeast mixture could improve the growth and vitality of yeast cells, and ultimately improve the fermentation degree [22]. Due to extensive applications of SODs in food and cosmetic industries as well as for various medicinal purposes, it is imperative to resolve the problem of low activity and stability. In this study, a novel *SaCSD1* from *S. alba* was cloned and the recombinant enzyme was expressed in *E. coli*. In addition, we analyzed the sequence and examined the activity and stability of the enzyme at different pHs, temperatures, and media. The expression of *SaCSD1* in different organs was detected. The altered expression of SaCSD1 in roots and leaves after treatment with different salt concentrations was also detected. Its relationship with the salt resistance properties of the plant was also explored preliminarily. The present work may lay the foundation for plant genetic engineering for stress resistance improvement, and the production of a novel Cu/Zn-SOD enzyme as an additive in healthcare products.

## 2. Results

### 2.1. Cloning of SaCSD1 and Sequence Analysis

**Figure 1:**
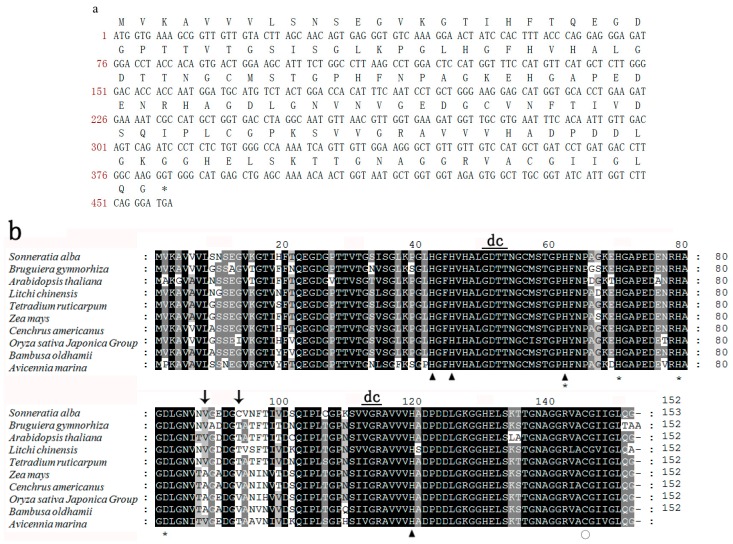
Sequence analysis of SaCSD1. (**a**) Coding sequence and amino acid sequence of SaCSD1; (**b**) Amino acid sequence alignment of CSDs for *Sonneratia alba* and other species. *Triangle* denotes copper ion binding sites, *asterisk* denotes zinc ion binding sites, *circle* denotes amino acids which form disulphide bonds, *arrow* indicates changes in the amino acids at the 89th and 94th positions between the plant species belonging to the *Poaceae* family and other families, and *dc* represents amino acids involved in dimer contact. Residues that are identical among the sequences are given a black background, and similar residues are in a gray background. The remaining residues are in a white background.

The sequence of *SaCSD1* was cloned for further analysis and characterization and its cDNA sequence was submitted to GenBank (Accession No. KF888631). The *SaCSD1* cDNA contained a 459-bp coding sequence, which encodes a protein of 152 amino acids (Figure 1a) with a predicted molecular weight of approximately 15.32 kDa and a pI of 5.78. We have concluded that *SaCSD1* has no signal peptide through the web site (http://www.cbs.dtu.dk/services/SignalP/). The result suggests that *SaCSD1* exists in cytosol.

Comparison of the *SaCSD1* deduced amino acid sequence showed high similarity (85%–90%) to the CSDs of other plant species, including several dicots and monocots (Table 1). Furthermore, multiple sequence alignment of these CSDs showed SaCSD1 contained potential binding sites for copper (H42, H45, H62, and H119) and zinc (H62, H70, H79, and D82) (Figure 1b). This result was in agreement with the previous studies [9,15].

**Table 1:** Amino acid sequence similarity of superoxide dismutases (CSDs) between *S. alba* and other plant species.

Species	GenBank Accession Number	Identity
*Tetradium ruticarpum*	AFF57842.1	90%
*Bruguiera gymnorhiza*	BAB78597.1	85%
*Litchi chinensis*	ABY65355.1	89%
*Avicennia marina*	ACA50531.1	84%
*Arabidopsis thaliana*	P24704.2	86%
*Oryza sativa*	AAA33917.1	85%
*Cenchrus americanus*	ABP65325.1	87%
*Zea mays*	NP001105704.1	85%
*Bambusa oldhamii*	ACX94084.1	87%

### 2.2. Expression, Purification, and Western Blot Analysis of SaCSD1

**Figure 2:**
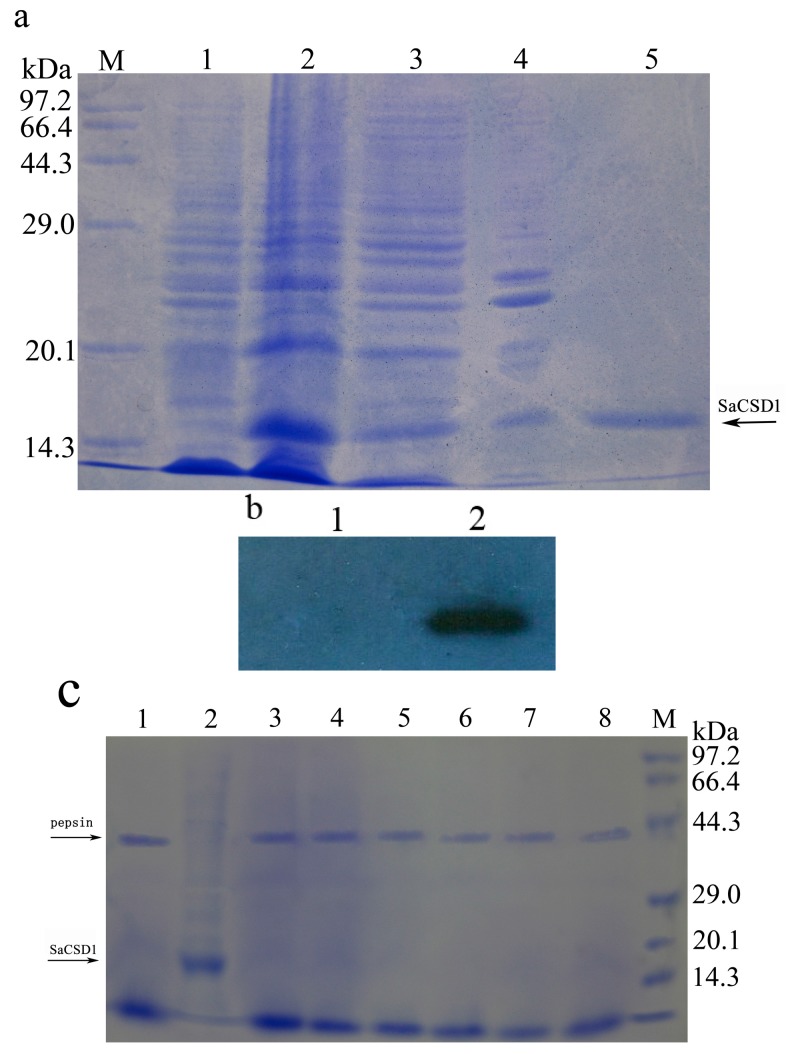
Expression, purification, Western Blot and pepsin digestion analysis of recombinant SaCSD1 protein. (**a**) Electrophoretogram of SaCSD1 protein. Lane M, protein marker; lane 1, uninduced *E. coli* lysate; lane 2, IPTG-induced *E. coli* lysate; lane 3, supernatant of the lysis after ultrasonication; lane 4, pellets of the lysis after ultrasonication; lane 5, the purified SaCSD1; (**b**) Western Blot detection of fusion protein with anti-His mAb. Lane 1, uninduced *E. coli* lysate; lane 2, the purified SaCSD1; (**c**) *In vitro* standardized pepsin digestion assay of SaCSD1 protein. Lane 1, the reaction without SaCSD1; lane 2, the reaction without pepsin; lane 3–8, the reaction in different times (0.5, 2, 5, 10, 20, 30 min); M, protein marker.

After electrophoresis, gray scanning analysis showed that the recombinant plasmid containing the *SaCSD1* gene was induced to express the protein with 30.6% yield in terms of total protein content. The expressed fusion protein was found in soluble form, and the purified protein was confirmed by SDS-PAGE electrophoresis, with a single band of approximately 16 kDa (Figure 2a). After affinity chromatography, recombinant SaCSD1 was obtained with 3.2-fold purification relative to the supernatant of the lysis after ultrasonication, and a specific activity of 2200 U/mg. Bovine CSD (diluted to 1000 U/mg) ordered from Sigma was used as the standard. Western Blot analysis using anti-His tag antibody confirmed the presence of the protein (Figure 2b).

### 2.3. Effects of pH, Temperature, Chemicals, and Salt on Activity of SaCSD1

**Figure 3:**
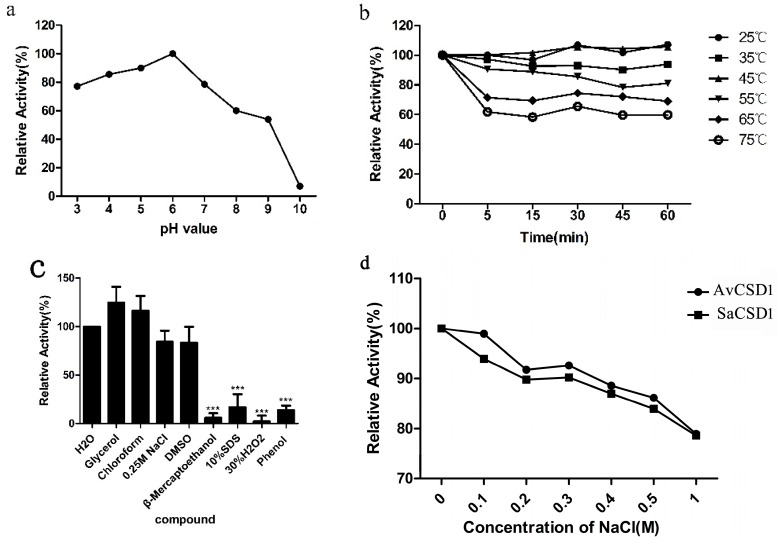
Effects of pH, temperature, chemicals, and salt on the activity of the purified SaCSD1. (**a**) pH stability of the purified SaCSD1, with the highest activity set as 100%; (**b**) Thermal stability of the purified SaCSD1. Activity of the enzyme without heat treatment was set as 100%; (**c**) Effects of various chemical compounds. Activity of the enzyme treated in H_2_O was set as the control and *** *p* < 0.001 means a significant difference from the control; (**d**) Effects of solutions with different salt concentrations on the activities of SaCSD1 and AvCSD1. Data are presented as the mean ± SD of three independent experiments.

The purified SaCSD1 had a functional pH range of 3.0–7.0 with more than 70% activity (highest at pH 6.0). The activity dropped rapidly at pH above 6.0 (Figure 3a), suggesting that SaCSD1 should be more stable in acidic conditions. No significant change in enzyme activity was observed when treated at 25–45 °C for up to 1 h. Even at 75 °C, 60% of enzyme activity was preserved after 1 h of treatment (Figure 3b), suggesting that the SaCSD1 is relatively thermostable. The enzymatic activity is increased about 20% after being treated in glycerol or chloroform, which is consistent with the findings of Xie [23]. The enzyme still retained 90% residual activity in DMSO. In addition, β-mercaptoethanol, SDS, H_2_O_2,_ and phenol have marked effects in reducing the activity of SaCSD1 (Figure 3c). We speculate that glycerol and chloroform may supply a hydrophobic environment that is conducive to better contact with the substrate. Both activity of SaCSD1 and AvCSD1 decrease with the increase of salt concentration, but SaCSD1 did not show significantly stronger activity on salt tolerance than AvCSD1 (Figure 3d).

### 2.4. Pepsin Digestion Characteristics of SaCSD1

The sensitivity of the recombinant SaCSD1 to the extreme acidity and action of pepsin in the digestive tract was then examined. The relative stability of the SaCSD1 to the extremes of pH and pepsin protease in the mammalian gastrointestinal (GI) tract was determined by the pepsin digestibility assay [24,25]. The pepsin digestibility assay showed that the SaCSD1 was degraded rapidly and completely after enzymatic treatment with pepsin. No full-length proteins were detectable even after 0.5 min of digestion (Figure 2c). The result demonstrated that the SaCSD1 protein was very susceptive to pepsin digestion.

### 2.5. Quantification of SaCSD1 Gene Expression in Different Organs and under Salt Stress

**Figure 4:**
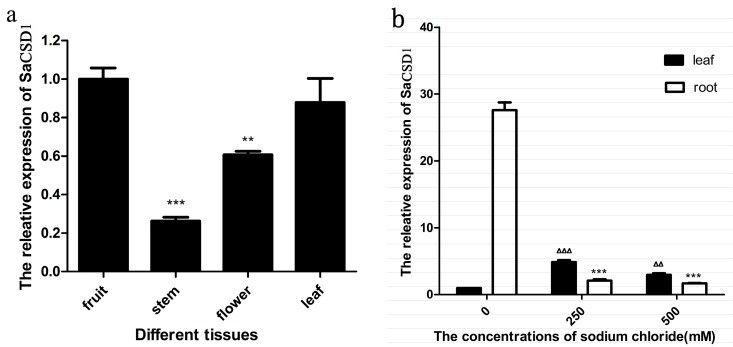
Real-time PCR analysis of the *SaCSD1* transcript. (**a**) Expression of *SaCSD1* in different organs. The highest expression (fruit) was set as 1.0 and ** *p* < 0.01 *** *p* < 0.001 means significant differences from it; (**b**) Expression of *SaCSD1* in roots and leaves treated with NaCl solutions. The blank group of leaves was set as 1.0 and, ^∆∆^ *p* < 0.01, ^∆∆∆^ *p* < 0.001 means significant differences from it, *** *p* < 0.001 means significant differences from the blank group of roots. *CSD1* Data are presented as the mean ± SD of three independent experiments.

In the expression assay, the highest transcription of the *SaCSD1* was detected in fruits and the lowest in stems. The expression level in leaves and flowers was intermediate (Figure 4a). After being treated in NaCl solution for seven days, the highest expression of the *SaCSD1* in the roots was detected in the 0 mM salinity, while both the 250 mM and 500 mM NaCl treatments showed significantly reduced expression (both approximately 6%, *p* < 0.001). In contrast, the lowest expression of the *SaCSD1* in the leaves was detected in the 0 mM NaCl treatment, and the expression was enhanced 4.8-fold (*p* < 0.001) in 250 mM salinity and 2.9-fold (*p* < 0.01) in 500 mM salinity (Figure 4b).

## 3. Discussion

Salinity has four main constraints on plant growth: (1) salinity inhibits plant growth and development by causing osmotic stress and thus restricting H_2_O absorption, hence the reduced assimilation, which in turn leads to (2) oxidative stress; salt stress also leads to (3) ionic toxicity; and (4) nutrient imbalance [26,27]. Extremely high salt concentrations or long-term exposure to a salinity condition induces the inhibition of enzyme activities and the over-accumulation of reactive oxygen species (ROS) such as superoxide radical (O^2−^), hydroxyl radical, and hydrogen peroxide (H_2_O_2_), resulting in metabolic disturbance, lipid peroxidation, chlorophyll breakdown, and so on [28,29]. ROS triggered by salt stress played an important role in adaptive responses, and ROS can promote signal transduction in adaptive responses at low concentrations, while they cause damage to macromolecules such as DNA, proteins, and membrane lipids at high concentrations [30,31]. SOD is the primary scavenger in the detoxification of active oxygen species in plants, and its function is to convert superoxide to H_2_O_2_ and O_2_. Superoxide dismutase (SOD) is also one of the crucial enzymes that protects cells against oxidative damage [32]. The roles of SODs under environmental stresses have been studied extensively [32]. It was reported that salt stress leads to a significant enhancement (88%) of total SOD activity in lentil roots due mainly to an enhancement in CSD isoforms, but leads to no significant increase in total SOD activity of leaf tissues [33]. NaCl stress could induce -SOD and CSD activities in thylakoids and in the stroma of *Pisum sativum* [34].

Studies in morphology, physiology, and ecology of mangroves have achieved much progress in the past two decades. Thanks to the development of genomic techniques, research at the genomic level has also been conducted on numerous species of mangroves recently [3]. Using Illumina sequencing technology, the transcriptome of *Sonneratia alba* has been uncovered [35]. Forty-five candidate salt-responsive genes (or gene families) were assumed in the transcriptome of *S. alba* and divided into four categories, including nine genes responsible for osmotic regulation, 12 genes that participated in ROS scavenging and detoxifying, 11 gene families related to salt uptake and transport, and 13 genes involved in signal transduction [35]. As one of the most salt-tolerant mangrove trees, *Sonneratia alba* grows in the low intertidal zones of tropical and subtropical coasts. The roots of *Sonneratia alba* are immersed in seawater, so extremely high saline intertidal habitats of *Sonneratia alba* are most likely associated with the roots. There are only two forms of SOD_S_ (CSD and Mn-SOD) that exist in roots. Mn-SOD only exists in mitochondria, while CSD is the most abundant SOD in plants, and has been mainly located in the cytosol. Cytosol is the major site of metabolism of cells, and it provides a stable microenvironment for the organelles. Combining a number of reports, it is likely that cytoplasmic *SaCSD1* may play an important role in adaptive responses to the seawater.

In the present study, we cloned and identified a cDNA sequence which encoded a novel CSD from *Sonneratia alba*, then expressed it in *E. coli*. The phylogenetic analysis reveals the presence of the conserved amino acid motifs essential for the enzyme function in the SaCSD1, which is responsible for the protein structure and catalytic function, and is even likely involved in the stabilizing conformation of CSD under harsh environmental conditions [36]. SaCSD1 shows high levels of homology to CSDs from other plants, suggesting that SaCSD1 might have the same function as that in other plants.

Real-time quantification PCR experiments showed that *SaCSD1* was expressed in all tested organs, with higher transcription in fruit and leaf and lower transcription in stem and flower. Since mangroves need to deal with various environmental stress factors such as high salinity, strong light intensity, and hypoxia, *SaCSD1* is likely to play an important role in protecting the fruit and leaf from ROS under these stress conditions. We found that the expression of *SaCSD1* is down-regulated in roots under salt stress, while it is up-regulated in leaves. We also found that the old leaves near the roots turned yellow during salt stress. Zhang *et al.* have demonstrated that the proportion of chloroplast SOD and the SOD activity in the halophyte *Suaeda salsa* increased with the increase of salt concentration or time [37]. Our results showed it was possible that growth and metabolism of the roots slow down after salt stress for seven days, while cellular metabolism and photosynthesis in the leaves accelerates in response to the high salt environment.

Our study showed that SaCSD1 was very stable at acidic and neutral pH and varying temperatures, which makes the enzyme suitable for use in industry. The effects of pH, temperature, and chemicals on SaCSD1 activity may lay a foundation of research for the various applications of SOD in healthcare products, cosmetics, food, and medicines. Its stable enzymatic activity in glycerol, DMSO, and chloroform may contribute to SaCSD1 protein preservation. The results of the pepsin digestibility assay suggested that SaCSD1 may not be suitable for oral products.

In conclusion, this is the first report of CSD gene cloning and expression from *S. alba*. Purification and characterization of SaCSD1 lay a foundation for future studies in the molecular mechanism of the adaptive evolution of mangroves. Furthermore, the biological functions and the potential therapeutic effects of SaCSD1 remain unclear. Therefore, further studies on SaCSD1 are required.

## 4. Materials and Methods

### 4.1. Materials

*Sma* I, Taq DNA polymerase High Fidelity Kit, CIAP, T4 DNA ligase and protein marker were purchased from Takara (Osaka, Japan). Isopropyl-β-D-thiogalactopyranoside (IPTG) and imidazole were ordered from TaKaRa (Dalian, China). Superoxide dismutase activity assay kit (WST-1 method) was purchased from Jiancheng (Nanjing, China). Mouse anti-His monoclonal antibody and Horseradish peroxidase (HRP)-labeled goat anti-mouse IgG were from TIANGEN (Beijing, China) and Proteintech (Chicago, IL, USA), respectively. Enhanced chemiluminescence (ECL) and detection reagent were obtained from Biyuntian (Haimen, China). Ni–NTA resin and SYBR Green PCR kit were purchased from QIAGEN (Hilden, Germany) and Takara (Osaka, Japan), respectively. Bovine Cu/Zn-SOD was ordered from Biyuntian (Haimen, China). All other reagents were analytical grade or better and commercially available.

### 4.2. RNA Preparation, cDNA Synthesis and Cloning

*Sonneratia alba* was collected from Qinglangang Mangrove Reserve, Wenchang, Hainan, China. Leaf (about 1 cm^2^) was ground to powder in liquid nitrogen. Total RNA was extracted and was further purified by using the modified CTAB method. Double-strand cDNA was generated using the M-MLV RTase cDNA Synthesis Kit (Takara) according to the instructions. For the amplification of SaCSD1, primers CSD1FP (5′-ATGGTGAAAGCGGTTGTTGTACT-3′) and CSD1RP (5′-TCATCCCTGAAGACCAATGATAC-3′) were designed based on the root transcriptome annotation of *Sonneratia alba* [8,35]. The coding region for putative mature *SaCSD1* was amplified by polymerase chain reaction (PCR). The PCR fragments were subjected to electrophoresis on 0.8% agarose gel and purified using a gel purification kit (Tiangen, Beijing, China). Then the purified product was ligated into the pMD-19T vector (Takara) and transformed into *E. coli* DH5α.

### 4.3. Nucleotide and Amino Acid Sequence Analysis of SaCSD1

The clone of *SaCSD1* was sequenced with the M13 forward and reverse primers. Vector NTI Advance 11 was used to analyze the nucleotide sequences, deduce amino acid sequences, and predict molecular mass and pI. Homologues of SaCSD1 amino acid sequences from other species were searched and obtained by BLASTP programs (http://blast.ncbi.nlm.nih.gov/Blast.cgi) and multiple sequence alignment was performed by Clustalx.

### 4.4. Cloning into the Expression Vector, Expression and Purification of SaCSD1

Plasmid pMD19T-*SaCSD1* was obtained by using a plasmid isolation kit (Tiangen). *SaCSD1* was amplified by PCR using the Pfu DNA Polymerase (Tiangen) and then ligated with pET-15b that was pretreated with *Sma* I and CIAP (Takara). The recombinant vector pET15b-*SaCSD1* was transformed into *E. coli* Rosetta-gami strain (Inovogen, Darmstadt, Germany) for expression. The transformant was cultivated at 37 °C in 200 mL Luria-Bertani (LB) medium containing 100 μg/mL ampicillin, 100 μg/mL kanamycin, and 100 μg/mL chloramphenicol while shaking (220 rpm) until OD_600_ approached 0.5–0.6. 0.5 Mm of Isopropyl β-Dithiogalactopyranoside (IPTG) which was then added. Thereafter the protein SaCSD1 was expressed at 30 °C for 8 h and then the bacterial cells were harvested by centrifugation at 10,000× *g* for 5 min. The cells were suspended again in 40 mL phosphate-buffered saline (PBS; pH 7.4) and an ultrasonic processor was used to break the cells and release the proteins. The cell lysates were centrifuged at 12,000 rpm for 2 min and then analyzed for protein expression by SDS–PAGE on 12% gels as described by Laemmli [38]. To purify this expressed SaCSD1, the supernatant was purified using a Ni-NTA His-Bind Resin. The purified protein was dialyzed for 24 h by using PBS buffer to remove imidazole.

### 4.5. Western Blot Analysis

Purified SaCSD1 was subjected to 12% SDS-PAGE and then electrophoretically transferred to polyvinylidene fluoride (PVDF) membrane at 200 mA for 20 min and then blocked with tris-buffered saline plus 0.1% Tween 20 (TBST) with 5% skim milk at 4 °C overnight. The blocked membrane was washed three times (5 min each time) in TBST and incubated with mouse anti-His IgG (diluted 1:1000) for 2 h at 25 °C. After washing three times with TBST, the membranes were probed with HRP-labeled goat anti-mouse IgG (diluted 1:3000) for 2 h at 25 °C. Lastly, the membrane was washed four times with TBST, exposed to enhanced chemiluminescence (ECL) plus detection reagent and visualized by chemiluminescence.

### 4.6. Determination of Protein Concentration and Activity Assay of SaCSD1

The protein concentration of SaCSD1 was assayed by the BCA method, using bovine serum albumin (BSA) as a reference substance. Superoxide dismutase activity assay kit (WST-1 method) was used to detect the SOD activity of SaCSD1. Bovine Cu/Zn SOD (diluted to 1000 U/mg) ordered from Sigma was used as the standard.

To investigate the optimum pH and temperature for SaCSD1, the effect of pH on dismutase activity of SaCSD1 was examined at 25 °C in the pH 3.0–10.0 for 2 h. Various pH buffers used were: 0.2 M Na_2_HPO_4_-0.1 M Citric Acid (pH 3.0–pH 8.0) and 0.05 M Glycine-NaOH buffer (pH 9.0–10.0). Thermal stability was examined by incubating the protein in the range of 25–75 °C for 0–60 min [39].

To assess the enzyme inhibition, the SaCSD1 protein solution (0.5 mg/mL) was mixed with the same volume of glycerol, chloroform, dimethylsulphoxide (DMSO), β-Mercaptoethanol, 10% sodium dodecyl sulfonate (SDS), 30% H_2_O_2_, and phenol, respectively. The enzymatic activity was examined after incubation at room temperature for two hours. Control with distilled water was treated in the same way as the test samples.

We next examined if the recombinant SaCSD1 protein from *S. alba* exhibited stronger activity on salt tolerance than other plants by comparing the activity of CSD proteins between *S. alba* and a terrestrial non–salt tolerant plant, *Amomum villosum*, under salt stress. The AvCSD1 protein was cloned and expressed by using the same method described above. The two CSD protein solutions (0.5 mg/mL) were mixed with the same volume of 0, 0.1, 0.2, 0.3, 0.4, 0.5, and 1 M NaCl solutions, respectively. After incubating at 37 °C for 4 h, we examined the enzymatic activity.

### 4.7. Pepsin Digestion Assay Conditions

Pepsin digestion assay was conducted according to the method of Wang [24]. Briefly, 380 µL of simulated gastric fluid (35 mM NaCl, 84 mM HCl, pH 2.0, 4000 U pepsin) was preheated at 37 °C and then 20 µL of SaCSD1 protein solution (5 mg/mL) was added. The mixture was kept in water bath at 37 °C. Samples of 40 µL were removed at 0.5, 2, 5, 10, 20, and 30 min after initiation of the incubation and immediately stored at 20 °C. Each 40 μL sample was quenched with 14 μL of 200 mM NaHCO_3_ (pH 11) and 14 μL 5× Laemmli buffer, then analyzed by 12% SDS-PAGE. The control samples (pepsin without test protein and reaction buffer with test protein but without pepsin) were analyzed in the same way.

### 4.8. Quantification of SaCSD1 Gene Expression by Real-Time PCR

To investigate the effects of salinity on the expression of SaCSD1, the seedlings of *S. alba* were planted in the greenhouse of Sun Yat-sen University under natural light conditions. After growing in the one-half Hoagland solutions for one month, these seedlings were transferred to one-half Hoagland solution with 0, 250, 500 mM NaCl for salt stress treatment, respectively. Tissues were harvested for RNA isolation after one week salt stress treatment. Quantitative reverse transcription-PCR in detection of the gene expression of *SaCSD1* was performed on an IQTM Multicolor Real-Time Detection System (BIO-RAD, Hercules, CA, USA). Then 2 µg total RNA from leaf, stem, flower and fruit was reversely transcribed, respectively. For samples under salt stress, root and leaf were used. Primers GAPDH RT-FP (5′-GTCCGTGGTTGACCTTACAGTGA-3′), GAPDH RT-RP (5′-CAATTCCAGCCTTAGCATCGAA-3′), SaCSD1 RT-FP (5′-CAACAGTGAGGGTGTCAAAGGAA-3′), SaCSD1 RT-RP (5′-CCAGCAGGATTGAAATGTGGTC-3′) were design for RT-qPCR. The reaction was performed as follows: 9 µL of diluted cDNA, 0.5 µL of each reverse and forward primers (10 µM), and 10 µL of SYBR Premix *Ex*Taq in 20 µL with one cycle of 95 °C for 30 s followed by 40 cycles of denaturation (5 s at 95 °C), and annealing (20 s at 60 °C). The primers of glyceraldehyde 3-phosphate dehydrogenase (GAPDH) of *S. alba* were used as the reference gene for data normalization and standard curve. The relative gene expression levels were reflected by relative quantification values, which were calculated using the 2^−ΔΔ*C*t^ method [40].

### 4.9. Statistical Analysis

All independent experiments were repeated three times. Experimental data were presented as means ± standard deviations (SD). The GraphPad Prism 5.0 software (San Diego, CA, USA) is used to make the Student′s *t*-tests or analysis of variance.

## Abbreviations

CSD: Cu/Zn-SOD, copper/zinc superoxide dismutase; ORF: open reading frame; SOD: superoxide dismutase; DMSO: dimethylsulfoxide; SDS: sodium dodecyl sulfonate; ROS: reactive oxidate species; RT-qPCR: real-time quantitative polymerase chain reaction; IPTG: Isopropyl Isopropyl d-beta-thiogalactoside; PBS: phosphate-buffered saline; TBST: tris-buffered saline plus 0.1% tween 20; ECL: enhanced chemiluminescence; BSA: bovine serum albumin; GAPDH: glyceraldehyde 3-phosphate dehydrogenase; SD: standard deviations.
